# Effects of universal adhesives on dentin matrix proteins, matrix metalloproteinases and cytokine release of human pulp cells

**DOI:** 10.1007/s10266-025-01107-3

**Published:** 2025-04-18

**Authors:** Bilge Ersöz, Numan Aydin, Elif Aybala Oktay, İrem Kübra Çal, Serpil Karaoğlanoğlu

**Affiliations:** Gulhane Faculty of Dentistry, Department of Restorative Dental Treatment, University of Health Sciences, 06018 Ankara, Turkey

**Keywords:** Adhesives, Cytotoxicity, Dentin matrix protein 1, Matrix metalloproteinases, Pulp cells

## Abstract

The potential toxicity of universal adhesives, which contain various monomers, solvents and fillers is a significant research topic. This study aims to investigate the toxicity and effects of universal adhesives on dentin matrix proteins (DMP-1), matrix metalloproteinases (MMP-2, MMP-8), tissue inhibitors of metalloproteinase-1 (TIMP-1), and cytokines [tumor necrosis factor-alpha (TNF-α) and interleukin-1 (IL-1)] in pulp cell lines. Six universal adhesives [Gluma Bond Universal (GBU), Prime&Bond Universal (PBU), Clearfil S3 Universal Bond (CS3UB), OptiBond Universal (OBU), G-2 Bond Universal (G2BU) and Bond Force II (BFII)] were assessed using MTT and ELISA cytotoxicity tests. The data obtained from MTT and ELISA tests were analyzed using two-way analysis of variance (ANOVA). The 1:1 extracts of BFII and GBU showed higher cell viability at 24 and 48 h compared to PBU, CS3B, OBU, and G2B adhesives (*p* < 0.001), and furthermore, the 1:1 extracts of GBU showed statistically the highest cell viability at 72 h (*p* < 0.001). The universal adhesives tested showed a significant decrease in TIMP-1 in pulp cells (*p* < 0.05), while TNF-α, IL-1, DMP-1, MMP-2 and MMP-8 levels did not change significantly. The tested adhesives exhibited varying degrees of cytotoxic effects depending on time and dose. The results indicate that the composition of these adhesives plays a crucial role in their cytotoxicity and impact on pulp cell viability. The amount and duration of adhesive application should be carefully regulated to maintain biocompatibility and ensure safe usage.

## Introduction

The development of dentin and enamel is regulated by interactions between epithelial–mesenchymal cells [1]. Dentinogenesis occurs through the formation of the organic extracellular dentin matrix (predentin), followed by the biomineralization of the predentin matrix to dentin. The organic components of predentin consist of Type I Collagen and non-cellular dentin matrix proteins (DMP-1) [2, 3]. DMP-1 and dentin sialophosphoproteins (DSPP) are non-collagen, non-cellular dentin matrices that regulate the dentin biomineralization of the predentin matrix [4].

Dentin also contains matrix metalloproteinases (MMPs): collagenase (MMP-8), gelatinases (MMP-2 and -9), stromelysin (MMP-3), enamelysin (MMP-20) [5]. MMPs and tissue inhibitors of metalloproteinases (TIMPs) play crucial roles in dentin formation, dental erosion, caries progression, pulpitis and hybrid zone degradation [6–8]*.* MMP-2, MMP-8 and MMP-9 have been identified in vitro in lesions of demineralized dentin [9]. The enzymatic activity of MMPs is regulated by TIMPs, which bind to the active sites of MMPs to inhibit their proteolytic activity. Under normal conditions, there is a balance between MMP and TIMP-1 levels. However, when this balance is disrupted, it can lead to various diseases. Increased MMP activity may cause conditions such as periodontitis, dental caries and pulpitis, while excessive TIMP-1 activation can lead to fibrosis and tissue hardening [10].

The tissue inhibitor TIMP-1 forms a compound with matrix metalloproteinases that avoids the decomposition of the extracellular matrix [11]. TIMP-1 has been shown to stimulate growth by inhibiting the apoptosis of cells associated with wound healing [12, 13]. Inflammatory mediators are released by cells exposed to adhesive materials [14]. Increased mediators have characteristic functions, such as involvement in the inflammatory process, differentiation and activation. For example, IL-6 is a pleomorphic cytokine that plays a particular role in the development and progression of inflammation. It is usually found in low doses in healthy teeth, but high rates are associated with irreversible pulpitis [15].

The natural healing potential of the pulp tissue is produced by the formation of repair dentin in the absence of bacterial contamination [16]. When pulp cells are exposed to low doses of TNF- α and IL-1 cytokines released by macrophages for a short period of time, the differentiation of pulp cells towards odontoblastic phenotype is facilitated. However, when pulp cells are exposed for a long time this differentiation is prevented [17–20].

Dental adhesives used in restorative treatments are classified as self-etch, total etch and selective etch according to their clinical application [21]. Dental adhesives can also be classified as single or two-stage systems according to the adhesion/clinical staging mechanisms. A two-stage self-etch system is a system in which the acid and monomer are in one bottle (acid + primer) and the adhesive is in another bottle. Single stage adhesives contain adhesive resin and acidic primers in a single bottle known as "all-in one." [22]. The universal single-stage adhesive group used in this study represents restorative procedures that have been simplified over time.

Universal adhesives are versatile and can be used both directly and indirectly to bond to different materials, such as enamel and dentin, composite resins, glass ceramics, zirconia and various metals [23]. The addition of silane to some of their compositions allows the silanization step to be skipped [24]. Furthermore, universal adhesives include other monomers that promote adhesion, such as dipentaerythritol pentaacrylate phosphoric acid ester (PENTA) and biphenyl dimethacrylate (BPDM), also hydrophobic decamethylene dimethacrylate (D3MA), hydrophilic hydroxyethyl methacrylate (HEMA), amphipathic bisphenol A-glycidyl methacrylate (bis-GMA), urethane dimethacrylate (UDMA) and triethylene glycol dimethacrylate (TEGDMA) [25]. The composition of universal adesives aims for better adhesion to various surfaces, while at the same time it can affect the biocompatibility of materials [26].

Different universal adhesives available on the market contain the mentioned monomers as well as components, such as acetone and ethanol, and they have varying pH levels and polymerization methods [25]. Studies have shown that these parameters affect the cytotoxicity of adhesives [27]. Although adhesive systems do not directly contact the pulp in vivo, the release of residual monomers can lead to acute toxic effects that may affect the pulp via dentinal tubules. Inadequately polymerized adhesives can cause pulpal inflammation and cell necrosis [26, 28].

The adhesives applied to the dentin surface create a hybrid layer between the filling and the dentin. This hybrid layer exhibits instability in aqueous environments due to the hydrolytic degradation of both resin and collagen fibrils [29–31]. It has been reported that collagen fibrils exposed within the hybrid layer can degrade if endogenous protease enzymes bound to the organic matrix of dentin are not protected by adhesive monomers [32, 33]. The matrix metalloproteinases in dentin have been reported to play an important role in the degradation of resin-infiltrated weak hybrid layers [34]. However, the proteinases remain structurally stable as long as the dentin matrix remains mineralised [35].

Although the presence of intrinsic proteolytic activity in teeth is clearly demonstrated, the effect of universal adhesives on proteolytical activity is unknown. The aim of this study is to study the effects of six universal adhesives with different contents on the cytotoxicity and release of DMP-1, MMP-2, MMP- 8, TIMP-1 and the cytokines TNF- α and IL-1.

## Materials and methods

In this study six different universal adhesives were used: Bond Force II (BFII), Clearfil S3 Universal Bond (CS3UB), G-2 Bond Universal (G2BU), Prime and Bond Universal (PBU), OptiBond Universal (OBU), Gluma Bond Universal (GBU) (Table 1).

**Table 1:** Contents of the universal adhesives used in the study

Universal adhesives	Contents	pH	Lot No.
Bond force II (Tokuyama Dental, Tsukuba, Japan)	Phosphoric acid monomer, Bis-GMA, TEGDMA, 2-Hydroxyethyl methacrylate HEMA, Camphorquinone, alcohol and purified water.	2.8	138E52
Clearfil S3 universal bond (Kuraray Noritake, Tokyo, Japan)	10-MDP, Bis-GMA, HEMA, colloidal silica, silane, sodium fluoride, camphoquinone, ethanol, water	2.5	000072
G2-Bond universal (GC, Tokyo, Japan)	Two-step universal adhesive Primer: 4-MET, 10-MDP, 10-MDTP, dimethacrylate monomer, acetone, water, initiators, fillers Adhesive: dimethacrylate monomer, Bis-GMA, filler, photoinitiator	1.5	1110251
Prime and bond universal (Dentsply Sirona, Konstanz Germany)	10-MDP, bisacrylamide monomers, PENTA, isopropanol, water, initiator, stabilizer	2.5	2112000750
Optibond universal (Kerr, Schaumburg, USA)	GPDM, glycerol dimethacrylate, HEMA, acetone, ethanol	2.3	9448958
Gluma bond universal (Kulzer, Hanau, Germany)	UDMA, MDP, 4-META, HEMA, acetone, water, photo initiators, stabilizers	1.5	M010058

### Preparation of samples

Six-well plate cell culture dishes, one for each adhesive, were used to prepare the adhesive extracts. Using a micropipette, 10 µl of each adhesive was placed of its designated well, creating six samples of each adhesive. Using a micropipette, 10 µl of each adhesive was placed into its designated well, creating six samples of each adhesive. In the two-step G2BU material, 10 µl of primer was additionally applied before the adhesive. All six samples were then diluted with pressurized air and polymerized at a power of 1200Mw/cm^2^ with an LED lighting device (O-Light, Woodpecker, Germany) for 10 s, according to the manufacturer’s recommendations. After polymerization, 1 ml of Dulbecco’s Modified Eagle Medium (DMEM) was added to the surface of each adhesive sample. All 36 samples were incubated at 37 °C for 24 h then strained through a 0.22 µm bacterial filter. A negative DMEM control group went through this same process.

### Cell culture

Human Dental Pulp Cells (PT 5025, LONZA) were used in this study. The pulp cells were added to 10% fetal bovine serum (FBS) and 1% penicillin streptomycin in DMEM. The cells were grown as monolayer cultures in sterile polystyrene T-75 flasks in a humidified incubator at 37°C with 5% CO_2_. Every 3 day DMEM were changed. After the cells covered the surface area of the flask, the enzyme tripsin-edta applied to the cell was removed from the surface of the flask and placed into 15 ml falcon tubes. The tubes were centrifugated at 1000 rpm for 5 min. The cells were then painted in tripan blue and counted on the Thoma Lam.

### Cytotoxicity test

Cell viability rate was determined using Thiazoly Blue Tetrazolium Bromide (MTT) analysis (MTT=3-(4,5-dimethylthiazol-2-yl)-5-(3-carboxymethoxyphenyl)-2-(4-sul-fophenyl)-2H-tetrazolium). For the MTT test, pulp cells (1 x 10^4^ cells/well) were placed in a 96-well plate. The cells were kept in an incubator containing 5% C0_2_ at 37 °C for 24 h to adhere the cells to the substrate. The prepared adhesive extracts were added to the cells in a 100 μl dilution of 1:1, 1:2 and 1:5. After incubating the adhesive extracts for 24, 48 and 72 h, 20 µl of the MTT solution (Sigma M2128 St. Louis, MO, USA) was dispensed into each of the 96 wells and incubated for 3–4 h. At the end of this period, the supernatant was aspirated without touching the cells. 100 μl of isopropyl alcohol was then added to each well, and the plate was incubated for 1 h at room temperature in a dark environment. Absorbance values of cell viabilities were measured in an automatic microplate spectrophotometer reader (Biotek Synergy HTX) at a wavelength of 570 nm. The percentage of cell vitality in the experimental groups was calculated as being 100% accepted in the control group.

### ELISA test

The amount of human dental pulp cells (PT-5025) released by cytokines was determined by Enzyme-Linked Immunosorbent Assay (ELISA). The prepared adhesive extracts were added to the cells at a diluation of 1:1 and kept in the incubator for 24 and 72 h. The media of the cells were collected after 24 and 72 h, separating the supernatants. Supernatants were tested in accordance with the manufacturer's instructions in the manual for each ELISA kit. This study used DMP-1 ELISA Kit, MMP-2 and 8 ELISA Kit, TIMP-1 ELISA Kit and TNF-a and IL-1 ELISA kits (SunRed Biological). The absorbance was converted into percentages, considering the control group (DMEM) as 100%.

### Statistical analysis

The data obtained from this study were subjected to statistical testing using the SPSS 22.00 Windows computer program (SPSS Inc, Chicago, IL, USA). In this study, MTT and ELISA test data were tested for normality distribution. The results of the MTT and ELISA tests were tested using two-way variance analysis (ANOVA). In the two-way ANOVA test, the variables were adhesive and dilution ratio. In the intergroup evaluation, the Tukey multiple comparison test was used. The level of statistical significance was *p* <0.05 for all adhesives.

## Results

**Fig. 1:**
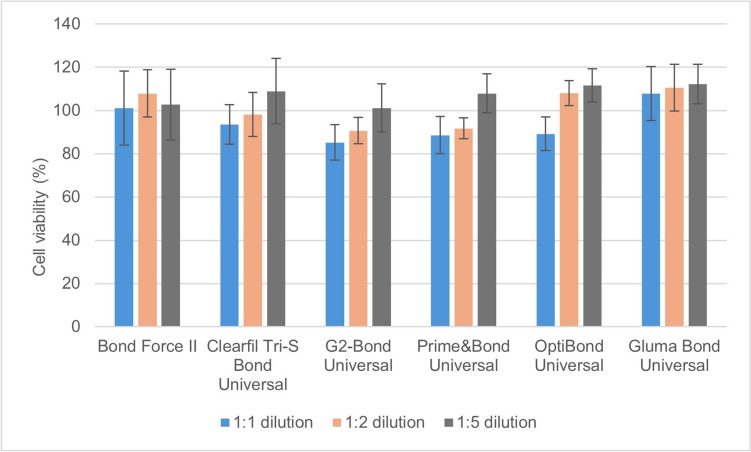
MTT test results of the adhesive extracts at different dilutions on pulp cell viability (%) after 24 h

**Fig. 2:**
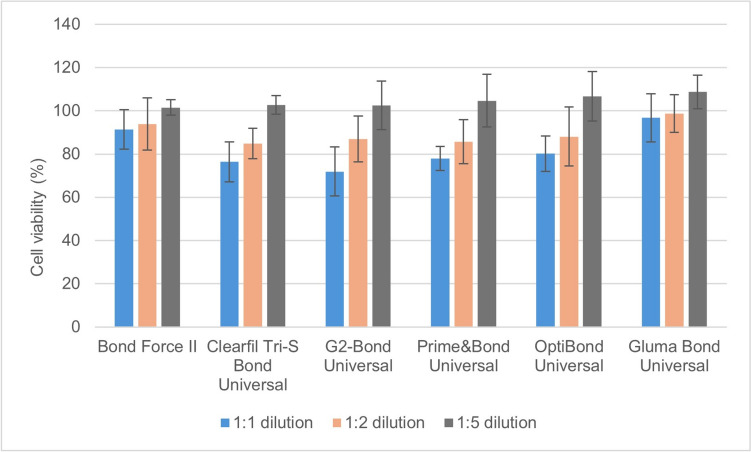
MTT test results of the adhesive extracts at different dilutions on pulp cell viability (%) after 48 h

**Fig. 3:**
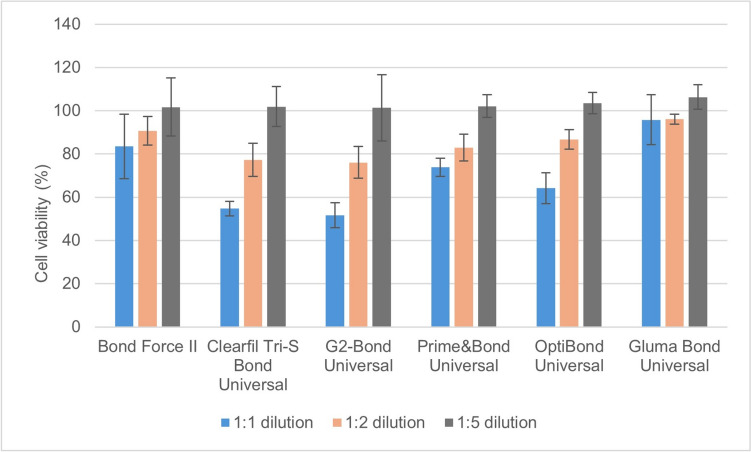
MTT test results of the adhesive extracts at different dilutions on pulp cell viability (%) after 72 h

Statistically significant differences were observed in cell viability at 24, 48 and 72 h after the application of the universal adhesive extracts (GBU, PBU, CS3B, OBU, G2B, BFII) on pulp cells (*p* = 0.01) (Figs. 1, 2, 3). The extracts of BFII and GBU in a 1:1 diluation did not affect the viability of pulp cells after 24 h (Fig. 1). Extracts of C3SB, G2BU, PBU and OBU at a 1:1 diluation decreased pulp cell viability. These same four adhesives extracts at 1:2 and 1:5 dilutions did not decrease pulpal cell viability (Table 2).

**Table 2:** MTT test results (% and standard deviation) of pulp cell viability of extracts of universal adhesives in different dilutions at the end of 24 h

Adhesives/dilution rate	1:1	1:2	1:5
Bond force II	101.1±17.1^a,A^	107.9±10.9^a,A^	102.7±16.4^ab,A^
Clearfil Tri-S bond universal	93.6±9.1^b,A^	98.1±10.2^b,A^	108.9±15.1^b,B^
G2-bond universal	85.2±8.2^b,A^	90.7±6.1^b,A^	101.2±11.1^a,A^
Prime and bond universal	88.6±8.6^b,A^	91.7±4.8^b,A^	107.9±9.1^b,B^
OptiBond universal	89.2±7.8^b,A^	108.1±5.8^a,B^	111.6±7.7^b,B^
Gluma bond universal	107.8±12.5^a,A^	110.5±10.8^a,A^	112.3±9.1^b,A^

*The statistical difference between adhesives is shown as a–b, and the statistical difference between dilutions is shown as A–B, (*p* <0.05)

All six universal adhesives used in this study at 1:1 and 1:2 dilutions decreased pulp cell viability at 48 h and 72 h (*p* = 0.01) (Figs. 2, 3). The highest decrease in pulp cell viability was seen in the C3SB, GBU, PBU and OBU adhesives. The universal adhesives at 1:5 dilution did not affect pulp cell viability at 48 h and 72 h (Tables 3, 4). The universal adhesives extracts of C3SB, GBU and OBU at 1:1 dilution showed pulp cell viability less than 70% after 72 h (Fig. 3). At 24 and 48 h, the 1:1 extracts of BFII and GBU caused statistically higher cell viability compared to PBU, CS3B, OBU, and G2B (p<0.001). At 72 h, the 1:1 extracts of GBU statistically reduced cell viability the least, while CS3B, OBU, and G2B significantly reduced cell viability (*p* < 0.001) (Table 3). At 72 h, the cell viability ranking of the 1:1 extracts of the adhesives was GBU > BFII > PBU > CS3B = OBU = G2B. Increasing the dilution ratio of the adhesive extracts (1:5) did not affect cell viability in pulp cells (Table 3).

**Table 3:** MTT test results (% and standard deviation) of pulp cell viability of extracts of universal adhesives in different dilutions at the end of 48 h

Adhesives/dilution rate	1:1	1:2	1:5
Bond force II	91.4±9.1^a,A^	93.9±12.1^a,A^	101.5±3.6^a,B^
Clearfil Tri-S bond universal	76.4±9.2^b,A^	84.9±7.1^a,A^	102.8±4.3^a,B^
G2-bond universal	71.9±11.3^b,A^	86.9±10.6^a,B^	102.5±11.2^a,C^
Prime and bond universal	77.9±5.6^b,A^	85.7±10.1^a,A^	104.7±12.1^a,B^
OptiBond universal	80.2±8.2^b,A^	88.1±13.6^a,A^	106.7±11.4^a,B^
Gluma bond universal	96.8±11.1^a,A^	98.7±8.7^b,A^	108.7±7.7^a,B^

*The statistical difference between adhesives is shown as a–b, and the statistical difference between dilutions is shown as A–C, (*p* <0.05)

**Table 4:** MTT test results (% and standard deviation) of pulp cell viability of extracts of universal adhesives in different dilutions at the end of 72 h

Adhesives/dilution rate	1:1	1:2	1:5
Bond force II	83.5±14.9^a,A^	90.7±6.6^a,A^	101.7±13.4^a,B^
Clearfil Tri-S bond universal	54.7±3.4^b,A^	77.3±7.6^b,B^	101.9±9.2^a,C^
G2-bond universal	51.7±5.8^b,A^	76.1±7.3^b,B^	101.3±15.3^a,C^
Prime and bond universal	73.8±4.3^c,A^	82.9±6.2^b,A^	102.1±5.3^a,B^
OptiBond universal	64.2±7.1^b,A^	86.7±4.6^b,B^	103.5±4.9^a,C^
Gluma bond universal	95.8±11.6^d,A^	96.1±2.3^c,B^	106.3±5.7^a,C^

*The statistical difference between adhesives is shown as a–d, and the statistical difference between dilutions is shown as A–C, (*p* <0.05)

All of the universal adhesive showed a statistically significant increase in TNF-α levels of pulp cells at 24 h (*p* < 0.05) and a statistically significant decrease in TNF-α levels at 72 h (p<0.05). There was no significant difference between the adhesives (*p* > 0.05). All of the universal adhesives showed an increase in IL-1 levels of pulp cells at 24 h and 72 h (*p* < 0.05). Although at 24 h, GBU showed the highest increase, there was no statistically significant difference between the adhesives at 72 h (*p* > 0.05). (Table 5)

**Table 5:** TNF-a and IL-1 elisa test results (mean and standard deviation) on pulp cells of extracts of universal adhesives at 1:1 dilution after 24 and 72 h

Adhesives	TNF-a (ng/ml)		IL-1 (pg/ml)	
	24 h	72 h	24 h	72 h
Bond force II	7.7±3.2^a^	5.9 ±9.1^a^	23.9±18.1^a^	12.2 ±4.3^a^
Clearfil Tri-S bond üniversal	10.2±25.1^a^	8.1 ±10.1^a^	18.4±3.1^ab^	11.8 ±6.9^a^
G2-bond universal	8.4±6.4^a^	6.6±9.9^a^	15.2±3.2^ab^	12.6±4.2^a^
Prime and bond universal	11.7±29.1^a^	5.1±3.6^a^	17.9±4.1^ab^	14.3±8.3^a^
OptiBond universal	9.7±7.5^a^	9.1±3.1^a^	18.6±5.2^ab^	18.0±4.1^a^
Gluma bond universal	9.6.6±7.1^a^	8.7±7.3^a^	31.3±14.4^c^	15.6±5.9^a^
Control (DMEM)	1.6±1.1^b^	21.3±9.4^b^	13.9±5.1^b^	10.3±5.3^a^

*Statistical difference between adhesives is shown as a–c, (*p* <0.05)

Although all of the universal adhesives showed a statistically significant increase in the amount of DMP-1 of pulp cells at 24 h (*p* < 0.05), the increase in the amount of DMP-1 was not statistically significant at 72 h (*p* >0.05). At the end of 24 h, GBU produced the highest increase in the DMP-1 amount of pulp cells. Universal adhesives decreased the amount of TIMP-1 after 24 and 72 h. There was no statistically significant difference between the adhesives (*p* > 0.05) (Table 6).

**Table 6:** DMP-1 and TIMP-1 elisa test results (mean and standard deviation) on pulp cells of extracts of universal adhesives at 1:1 dilution after 24 and 72 h

Adhesives	DMP-1 (ng/ml)		TIMP-1 (ng/ml)	
	24 h	72 h	24 h	72 h
Bond force II	6.7±3.4^a^	12.5 ±3.3^a^	17.4±7.6^a^	18.9 ±3.6^a^
Clearfil Tri-S bond universal	11.4±3.5^b^	10.3 ±1.4^a^	24.9±6.9^a^	23.2 ±5.9^a^
G2-bond universal	17.5±5.8^c^	11.4±1.1^a^	20.9±3.1^a^	16.7±4.3^a^
Prime and bond universal	9.6±7.7^ab^	12.8±1.6^a^	18.8±7.1^a^	16.5±1.6^a^
OptiBond universal	10.1±1.9^ab^	12.2±2.2^a^	16.2±4.2^a^	21.4±7.4^a^
Gluma bond universal	12.1±2.0^ab^	13.5±0.5^a^	21.6±4.2a	21.5±5.9^a^
Control (DMEM)	2.70±2.5^d^	11.3±1.6^a^	38.1±12.8^b^	26.7±9.4^b^

*Statistical difference between adhesives is shown as a–d, (*p* <0.05)

Compared to the control group, none of the universal adhesives showed a statistically significant increase in matrix metalloproteinases of pulp cells at 24 and 72 h (Table 7). BFII showed a higher increase in the amount of MMP-8 at 24 and 72 h. PBU and OBU showed a decrease in MMP-8 levels at 72 h. Although all adhesives used in this study showed an increase in the amount of MMP-2 in pulp cells at 24 and 72 h, it was not statistically significant when compared to the control group ( p > 0.05).

**Table 7:** Elisa test results of matrix metalloproteinases on pulp cells at the end of 24 and 72 h of extracts of universal adhesives at 1:1 dilution (mean and standard deviation)

Adhesives/matrix metalloproteinases	MMP-8 (ng/ml)		MMP-2 (ng/ml)	
	24 h	72 h	24 h	72 h
Bond Force II	2.48±0.5^a^	2.36 ±0.8^a^	0.90±0.4^a^	0.98 ±0.3^a^
Clearfil Tri-S bond universal	2.38±0.3^a^	2.23 ±0.3^a^	1.16±0.3^a^	1.2 ±0.4^a^
G2-bond universal	2.33±0.3^a^	2.20±0.3^a^	1.04±0.5^a^	1.17±0.2^a^
Prime and bond universal	2.27±0.5^a^	1.87±0.4^b^	1.18±0.5^a^	1.05±0.3^a^
OptiBond universal	2.24±0.2^a^	1.98±0.4^b^	0.90±0.1^a^	1.15±0.4^a^
Gluma bond universal	2.32±0.2^a^	2.38±0.3^a^	1.16±0.6^a^	1.05±0.2^a^
Control (DMEM)	2.20±0.2^a^	2.42±0.6^a^	0.80±0.5^a^	0.96±0.4^a^

*Statistical difference between adhesives is shown as a–b, (*p* <0.05)

## Discussion

Cytotoxicity tests are a fundamental step in evaluating the biocompatibility of a material. This study was conducted to assess the toxicity of universal adhesives with different chemical compositions to human pulp cells.

Animal experiments and cell culture tests are commonly used to assess the cytotoxicity of dental materials. However, animal experiments are controversial, time-consuming, and costly methods [36]. Due to their advantages, such as low cost, controllability, and ease of application, cell culture tests have been increasingly used as an alternative to animal experiments [37]. In vitro cell culture testing is widely utilized in restorative dentistry to evaluate the biocompatibility of dental materials [38]. Various in vitro testing methods such as indirect and extract tests are used to evaluate the cytotoxic effects of biomaterials in cell culture studies [39, 40]. The most widely method used for these tests is the extract method. This method allows for the simulation of the clinical environment, since the adhesives are not in direct contact with the cells. Pagano et al. reported that the extract test method was more suitable for evaluating adhesives [41]. Therefore, the extract test method was chosen for this study.

The MTT test is an objective method for determining cell vitality and is applied to all cells that are metabolically active and, therefore, alive [42]. It is frequently cited in the literature as the chosen method to test the cytotoxicity of dental adhesives and other dental materials [43, 44]. In this study, the MTT test was used to detect cell vitality.

In addition to the composition of adhesives, pH is an important factor that can affect cytotoxicity. The pH values of universal adhesives are categorized into four groups: moderately strong (<1), strong (1,5), mild (>2), and ultra-mild (2,5) [45]. In this study, universal adhesives with a strong pH (GBU and G2BU, pH: 1.5), mild pH (OBU, pH: 2,3), and ultra-mild pH (BFII, pH: 2,8; C3SUB, pH: 2,5; PBU, pH: 2,5) were used. According to previous studies, the toxicity of adhesives has been reported to increase with higher acidic levels. However, it was not possible to evaluate the sole effect of pH on cytotoxicity in this study [46]. In addition, when the results of the study were examined, it was observed that as dilution increased, cytotoxic effects decreased, which may be related to the reduction in acidity. However, further studies are needed to investigate this topic in more detail.

It has been reported that parameters such as the type of curing device tip, light spectrum, and intensity used in the polymerization of dental adhesives affect their cytotoxic properties [47]. Polymerization with low-power light may prevent the complete conversion of monomers into polymers, leading to an increase in residual monomer content and a risk of leakage into surrounding tissues [48]. Therefore, the characteristics of the light-curing devices used during polymerization are of great importance. In a study conducted by Ergün et al., the cytotoxic effects of LED and halogen light sources on three different adhesive systems were examined [47]. Another study evaluated the cytotoxicity of an adhesive system on L929 mouse fibroblast cells using both LED light-curing devices and chemical polymerization methods [28]. In both studies in the literature, it was determined that the number of cells in the experimental group polymerized with an LED light device was higher, indicating lower cytotoxic effects [28]. Therefore, in this study, an LED light source with a power of 1200 MW/cm^2^ was applied to ensure proper polymerization and light intensity.

Bis-GMA, UDMA, TEGDMA and HEMA monomers in adhesives are associated with cytotoxicity [49, 50]. Among the monomers used in adhesives, Bis-GMA has relatively high cytotoxicity, but due to its high molecular weight it has a low capacity to penetrate dentine [51]. Bis-GMA has been documented to induce cytotoxicity in pulp cells by stimulating prostanoid production, potentially leading to inflammation or pulpal necrosis as a consequence of increased reactive oxygen species (ROS) generation [52]. On the other hand, HEMA has the ability to rapidly diffuse through dentin and can cause toxicity in the pulp tissue of cells [51]. It can also suppress the growth of many cell types and causes a delay in primary fibroblast cell cycle progression, especially by increasing the formation of reactive oxygen species (ROS) [53]. If ranking these monomers by toxicity, the most toxic is Bis-GMA, followed by UDMA, TEGDMA and HEMA, the least toxic [28]. The release of residual monomers, such as HEMA, Bis-GMA, and TEGDMA, which cause toxicity, is directly influenced by the degree of monomer conversion that occurs during adhesive polymerization [54]. It is known that current adhesive systems cannot hermetically seal deep dentin, and therefore, these residual monomers and other components of adhesive systems may penetrate through the dentinal tubules into the dental pulp chamber, potentially causing irreversible damage to the cellular tissue [55].

In addition to the cytotoxicity of traditional monomers found in dental adhesives, the cytotoxicity of acidic functional monomers is also important. Universal adhesives contain different acidic functional monomers, such as 10-methacryloyloxydecyl dihydrogenphosphate (10-MDP) and 4-methacryloyloxyethyl trimellianhydride (4-META). 10-MDP is the most commonly used acidic monomer in universal adhesives. It contains a dihydrogen phosphate group that causes tooth corrosion and a methacrylate group for cross-binding with other resin monomers [56]. 10-MDP promotes an inflammatory response and suppresses odontoblastic differentiation of human pulp cells [57]. 10-MDP also stops odontoblastic differentiation, but it is also thought to easily bind to calcium made by odontoblast-like cells and stop mineralization directly [58].

Although 4-META, another functional monomer used in adhesives, has been shown to have a high level of biocompatibility with dental pulp cells, studies investigating its cytotoxicity are limited [59, 60]. In addition, the interaction between monomers is also very important in terms of toxicity, as it has been shown that synergistic effects between monomers may develop when combinations of 4-META with TEGDMA, UDMA or especially Bis-GMA are tested [28].

The results of *in vitro* studies on adhesives provide useful information, although this information is not always interrelated. Many *in vitro* studies have reported different levels of cytotoxic effects of the adhesive system [40, 41, 61]. Testing nine adhesives, Tu and et al. [62] examined their cytotoxic effects on oral epithelial cells. Özen and et al. [63] noted that four different adhesives exhibited cytotoxicity to gingival fibroblast cells after 72 h. Demirel et al. [40] noted that the 1:1 adhesives extracts showed 60–90% cell vitality on L929 cells after 72 h. Yavuz and Surmelioglu reported that the cytotoxicity of adhesives varies depending on the exposure time [64].

The universal adhesives used in this study showed a cytotoxic effect after 48 h and 72 h, although they did not exhibit a 24-h cytotoxic effect. These results are similar to the results of studies discussed in the literature [40, 63]. The toxic effects of universal adhesives decreased as the dilution rate increased. Among the universal adhesives, those containing 4-META (GBU) affected pulpal cell viability less than those without 4-META. There were no statistically significant differences in composition between the universal adhesives containing 4-MET (G2BU), 10-MDP (C3SB, G2BU, PBU), 10-MDTP (G2BU) and GPDM (OBU) at all three times.

Inflammatory mediators are released by cells exposed to adhesive materials [14]. Increased mediators have characteristic functions, such as involvement in the inflammatory process, differentiation and activation. For example, IL-6 is a pleomorphic cytokine that plays a particular role in the development and progression of inflammation. It is usually found in low doses in healthy teeth, but high rates are associated with irreversible pulpitis [15]. In this study, the universal adhesives caused an increase in the release of TNF- α and IL-1 at the end of the first 24 h, but the release of TNF-α and IL-1 decreased in the control group after 72 h. The inflammatory response observed at the end of 24 h is thought to be caused by the monomers present in the adhesives, including Bis-GMA, TEGDMA, UDMA, HEMA, 10-MDP, 10-MDTP, 4-MET, and PENTA. In addition, among the tested adhesives, GBU induced the highest increase in TNF-α and IL-1 release at the end of the first 24 h. The low pH (1.5) and HEMA content of GBU can be considered potential factors contributing to the severity of the inflammatory response.

Monomers, such as TEGDMA, that leak from resin-based restorative materials have been shown to activate and induce the production of MMP-2, MMP-8 and MMP-9 in pulp cells [65]. In the studies of Lehmann N et al. and Van Meerbeck B et al., an increase in pulp MMP-2 and MMP-9 expression was found in self-etch adhesive application [66, 67]. Similarly, Orsini et al. observed an increase in MMP-2 expression after applying it directly onto human pulp cells, although using different adhesives than those used in this study [68]. In this study BFII containing TEG–DMA increased MMP-2 and MMP-8 levels, although statistically not significant, while PBU and OBU decreased MMP-8 levels. This could be due to the pH levels of PBU (2,5) and OBU (2,3).

Matrix metalloproteinases (MMPs), which are among the most important proteolytic enzymes of the extracellular matrix (ECM), have the ability to degrade all protein-based ECM components. MMP activity plays a role in various oral pathologies, including dental erosion, tooth decay, pulpitis, periapical periodontitis, and oral cancer invasion and metastasis [6–8]. In addition, MMP activity is involved in odontogenesis and tissue regeneration, and its dysregulation can lead to inadequate enamel mineralization or gingival overgrowth [9]. Therefore, fluctuations in MMP levels can disrupt the balance between degradation and repair processes. In particular, the direct application of adhesives to the pulpal wall in deep cavities may lead to pulpal pathologies and, consequently, periapical inflammation.

In this study, universal adhesives showed an increase in TNF- α and IL-1 levels in pulp cells after 24 h. However, at the end of 72 h this increase was not statistically significant. Universal adhesives also increased the amount of DMP-1 in pulp cells after 24 h, but did not make a statistically significant increase after 72 h. This increase in the DMP-1 level of pulpal cells at the end of 24 h is considered to be related to the increase in the amount of cytokines (TNF-a and IL-1) released from pulpal cells after application of universal adhesives.

Yoshioka et al. reported on the critical molecules involved in tertiary dentin formation using a comprehensive gene expression analysis on pulp cells [69]. According to the results, hybridization reveals that TIMP-1 is expressed by pulpal cells. It is also noted that TIMP-1 plays a role in the wound healing process in pulp tissue and could be a biological target for regenerative therapy [61].

In this study, universal adhesives did not produce a statistically significant increase in the amount of MMP-2 and MMP-8 in pulp cells. They did, however, cause a decrease of the tissue inhibitor TIMP-1 in the pulp cells. Although this decrease is not a statistically significant difference, it is estimated to be associated with an increase in MMP-2 and MMP-8.

The level of MMP, responsible for proteolytic activity, remained unchanged; however, the level of TIMP-1, which is responsible for tissue regeneration and the suppression of MMP’s proteolytic activity, decreased. This may hinder the proper healing of the dentin–pulp complex, negatively affecting reparative dentin formation and tissue regeneration. In addition, insufficient TIMP-1 levels may contribute to inflammation and excessive tissue remodeling, which could impair pulp tissue healing and long-term stability. Therefore, the decrease in TIMP-1 may indicate a disruption in the regulation of MMP activity, potentially leading to an impaired dentin-pulp healing process.

The limitations of this study are that it took place in an *in vitro* environment and was carried out on pulp cells for a short length of time. After the adhesives are applied to the tooth, they remain longer in the oral environment than the time tested in this study.

Within the limitations of this *in vitro* study, universal adhesives showed time- and dose-dependent cytotoxic effects on human pulp cells. Universal adhesives increased the amount of the DMP-1 protein in the pulp cells within the first 24 h, but did not affect it after 72 h. The level of the tissue inhibitor TIMP-1 in the pulp cells was reduced by the universal adhesives used, whereas the levels of MMP-2 and MMP-8 were unaffected.

Therefore, the application techniques of universal adhesives should be carefully reviewed to minimize cytotoxicity risks. In deep dentin cases with a thin dentin barrier, adhesives should be applied with caution, while their use should be avoided in areas with direct contact with the dental pulp. In addition, to prevent residual monomers and potential cytotoxic effects, a high-quality light source should be used to enhance polymerization efficiency. The dosage and application time of adhesives should also be meticulously controlled to ensure their biocompatibility and safe use.

## Data Availability

The data sets generated and/or analyzed during the current study are available from the corresponding author on reasonable request.
